# Diagnosis and Emerging Biomarkers of Cystic Fibrosis-Related Kidney Disease (CFKD)

**DOI:** 10.3390/jcm14155585

**Published:** 2025-08-07

**Authors:** Hayrettin Yavuz, Manish Kumar, Himanshu Ballav Goswami, Uta Erdbrügger, William Thomas Harris, Sladjana Skopelja-Gardner, Martha Graber, Agnieszka Swiatecka-Urban

**Affiliations:** 1 Department of Pediatrics, University of Virginia School of Medicine, Charlottesville, VA 22903, USA; eeu9ng@virginia.edu; 2 Department of Pediatrics, Heersink School of Medicine, University of Alabama at Birmingham, Birmingham, AL 35233, USA; manishkumar@uabmc.edu (M.K.); wtharris@uabmc.edu (W.T.H.); 3 Department of Microbiology and Immunology, Dartmouth Geisel School of Medicine, Lebanon, NH 03756, USA; himanshu.ballav.goswami.gr@dartmouth.edu (H.B.G.); sladjana.skopelja-gardner@dartmouth.edu (S.S.-G.); 4 Department of Medicine, University of Virginia School of Medicine, Charlottesville, VA 22903, USA; ue2u@virginia.edu; 5 Department of Medicine, Dartmouth Geisel School of Medicine, Lebanon, NH 03756, USA; martha.graber@dartmouth.edu

**Keywords:** cystic fibrosis, kidney, CFTR, biomarkers, CKD

## Abstract

As people with cystic fibrosis (PwCF) live longer, kidney disease is emerging as a significant comorbidity that is increasingly linked to cardiovascular complications and progression to end-stage kidney disease. In our recent review, we proposed the unifying term CF-related kidney disease (CFKD) to encompass the spectrum of kidney dysfunction observed in this population. Early detection of kidney injury is critical for improving long-term outcomes, yet remains challenging due to the limited sensitivity of conventional laboratory tests, particularly in individuals with altered muscle mass and unique CF pathophysiology. Emerging approaches, including novel blood and urinary biomarkers, urinary extracellular vesicles, and genetic risk profiling, offer promising avenues for identifying subclinical kidney damage. When integrated with machine learning algorithms, these tools may enable the development of personalized risk stratification models and targeted therapeutic strategies. This precision medicine approach has the potential to transform kidney disease management in PwCF, shifting care from reactive treatment of late-stage disease to proactive monitoring and early intervention.

## 1. Introduction

Cystic Fibrosis (CF) is a genetic disorder caused by mutations in the CF Transmembrane Conductance Regulator (*CFTR*) gene [1]. Since the discovery and clinical use of highly effective modulator therapy (HEMT), which improves mutant CFTR protein expression and/or function, life expectancy for people with CF (PwCF) has increased significantly and resulted in substantial improvements in lung function and quality of life [2,3]. Chronic kidney disease (CKD) has emerged as a notable morbidity in the aging population of PwCF [4,5]. CF-related kidney disease (CFKD) has been proposed as a term for kidney dysfunction affecting PwCF [6,7]. In addition to renal morbidity, CKD is an identified risk factor for cardiovascular events, subsequent end-stage kidney disease (ESKD), and death [8]. The known risk factors for acute kidney injury (AKI) in PwCF include nephrotoxic medications (e.g., aminoglycosides, non-steroidal anti-inflammatory drugs), severe pulmonary infections, systemic inflammation, hypoxia, and oxidative stress [9,10,11]. AKI is frequently undetected by conventional assays and may increase the risk of CKD [12]. Evidence-based interventions such as lifestyle modifications and blood pressure control, as well as monitoring medication use during the early stages of kidney injury, may prevent or delay CKD [13,14].

CFTR, abundantly expressed in most segments of the renal tubular epithelium, is implicated in diverse functions that differ from those in the lung [6,15,16,17]. In the proximal tubules of the kidney, CFTR regulates vesicular acidification and the endocytic uptake of low-molecular-weight proteins. In the collecting duct, it plays a role in bicarbonate secretion (reviewed in Hart et al., 2025) [6]. The mechanisms of CFKD are incompletely understood and may include factors associated with CFTR dysfunction in the lung and other organs, including the kidney, cumulative treatment side effects combined with an age-related kidney function decline, as well as genetic factors [6,18,19]. Here, we review conventional and emerging biomarkers for early detection of kidney injury and dysfunction, with a focus on PwCF. To guide future research on CF-specific biomarkers of kidney dysfunction, we describe genetic modifiers associated with worse outcomes in CF and emphasize their potential as CFKD contributors.

To identify relevant studies for this review, we conducted a literature search using the PubMed database. The search included combinations of the following keywords: “Cystic fibrosis,” “kidney injury,” “biomarkers,” “chronic kidney disease (CKD),” “acute kidney disease (AKD),” “CF-related kidney disease (CFKD),” and “CFTR.” We included original research articles, reviews, and clinical studies that focused on biomarkers associated with kidney disease in people with cystic fibrosis, as well as in the general population to offer a comprehensive view of the current biomarker landscape. Studies that did not address kidney disease or biomarker relevance were excluded. For emerging biomarkers, we focused on studies published within the past 10 years (2014–2024) to ensure relevance to current research and clinical developments. Additional references were included when necessary to provide background or foundational knowledge.

## 2. Conventional Laboratory Tests for Kidney Function and the Detection of Kidney Disease

Several laboratory tests performed on blood and urine specimens are typically used in clinical practice to evaluate kidney function. Most tests are not disease-specific and provide only an imperfect estimate of kidney function. Results of several of these tests have to be interpreted together to better understand kidney health and the potential etiology of dysfunction. For this reason, conventional laboratory tests do not meet the criteria of a biomarker, which is defined as a measurable and objectively evaluated characteristic that indicates normal physiological or pathological processes [20].

### 2.1. Serum Creatinine Concentration and Glomerular Filtration Rate (eGFR)

Serum creatinine concentration and glomerular filtration rate (GFR) are commonly used measurements in clinical practice to estimate kidney function. GFR reflects the kidney’s ability to filter blood, but its direct measurement is often impractical in clinical settings. Serum creatinine concentration is used as a surrogate measure to estimate GFR (eGFR; normal range 90–120 mL/min/1.73 m^2^). Creatinine, a by-product of muscle metabolism, is filtered by the glomeruli. It is not an ideal substance for measuring GFR because it is also secreted by the proximal tubule, leading to an overestimation of GFR by 10–20% [21]. Creatinine levels are influenced by age, sex, muscle mass, nutritional and hydration status, diet, and the use of medications. Its production decreases during sepsis [22].

Serum creatinine is primarily measured using the Jaffe reaction, enzymatic assays, isotope dilution mass spectrometry (IDMS), or high-performance liquid chromatography (HPLC). The Jaffe reaction is a simple and cost-effective colorimetric assay, but it is also limited by susceptibility to interference [23]. Enzymatic methods utilize specific enzymes, providing greater specificity and less interference than the Jaffe method, although they are more expensive [24]. IDMS is considered the gold standard for creatinine measurement, providing high precision and serving as a reference method for standardization, but its high cost and limited availability restrict its routine clinical use [25]. HPLC separates creatinine from other serum components before detection, minimizing interference. While it is highly specific and accurate, it is time-consuming and requires specialized equipment [26]. GFR estimation methods rely on the assumption of a steady state—a condition in which the rate of creatinine production by the body equals the rate of its clearance by the kidneys. Creatinine reflects eGFR during the steady state, and the utility of serum creatinine declines as kidney disease progresses [27]. In response to injury, the kidney deploys compensatory mechanisms, leading to hyperfiltration through functioning glomeruli that maintain normal or even elevated eGFR, thereby masking the progressive loss of nephrons. Serum creatinine levels only begin to increase after significant kidney damage has occurred, with renal function declining by as much as 50% before noticeable changes in creatinine are detected [28]. Therefore, the correlation between kidney function and eGFR is non-linear; eGFR does not accurately predict early kidney disease or define the mechanisms of injury [29]. An overestimation of eGFR due to creatinine secretion by the kidney proximal tubule further complicates measurements.

### 2.2. Serum Cystatin C

Cystatin C, a substance released by all nucleated cells, has been used to measure eGFR (recommendations from the National Kidney Foundation & American Society of Nephrology NKF-ASN Task Force) [30]. It is particularly useful when combined with serum creatinine measurements [31]. Various eGFR formulas exist, based on measurements of serum creatinine and/or Cystatin C, age groups, sex, and population characteristics. Some of the most commonly used formulas include those from the Chronic Kidney Disease in Children (CKiD) Study and the CKD-EPI, which are tailored to specific groups for more accurate estimation of kidney function. For example, the CKiD U25 eGFR formula is specifically designed for children and young adults aged 1–25 years. Other formulas are optimized for specific populations, including those with CKD or older adults (Table 1) [32,33,34,35,36]. Two independent studies concluded that cystatin C clearance outperforms eGFR in PwCF [37,38]. However, none of the current formulas for eGFR are validated in PwCF, and thus the correlation with GFR in PwCF is unclear [39].

**Table 1:** eGFR formulas based on age groups and sex.

eGFR Formula	Population	Age Group	Characteristics
CKiD U25 eGFR	CKiD	1–25 years	Age, sex, height, serum creatinine, and serum Cystatin C [32,33]
CKD-EPI 2021 (Creatinine)	General adult population	≥18 years	Age, sex, and serum creatinine [32]
MDRD Study	CKD	≥18 years	Age, sex, race, and serum creatinine [34]
Schwartz Formula	Pediatric population (general)	1–18 years	Height and serum creatinine [35]
CKD-EPI 2012 (Creatinine-Cystatin C)	General adult population	≥18 years	Age, sex, race, serum creatinine, and serum Cystatin C [36]

CKiD, chronic kidney disease in children; eGFR, estimated glomerular filtration rate; CKD-EPI, chronic kidney disease epidemiology collaboration; MDRD, modification of diet in renal disease; CKD, chronic kidney disease.

### 2.3. Urine Protein Composition and Concentration

Urine protein composition and concentration are used to assess glomerular and tubular integrity. Urinary albumin excretion is typically classified into three categories: normoalbuminuria (<30 mg/day or UACR < 30 mg/g), microalbuminuria (30–300 mg/day or UACR 30–300 mg/g), and macroalbuminuria (>300 mg/day or UACR > 300 mg/g) [40]. Excessive urinary albumin concentration (macroalbuminuria) indicates glomerular damage. By contrast, tubular damage is suggested by elevated concentrations of urinary low molecular weight (LMW; molecular weight < 40 kDa) proteins, such as beta-2 microglobulin (β2MG) and retinol-binding protein (RBP) in the presence of normoalbuminuria [41]. Urinary LMW proteins are typically measured using various biochemical and electrophoretic techniques, including enzyme-linked immunosorbent assay (ELISA), sodium dodecyl sulfate-polyacrylamide gel electrophoresis (SDS-PAGE), liquid chromatography-tandem mass spectrometry (LC-MS), and Urine Protein Electrophoresis (UPEP), which is a clinical laboratory test [42,43,44].

Microalbuminuria can be at least partially mitigated by tubular reabsorption of albumin. This process exerts metabolic stress on the renal proximal tubule where the reabsorption occurs, leading to tubular damage, tubulointerstitial fibrosis, and CKD progression over months to years. For all these reasons, worsening proteinuria is a test of established kidney injury and its progression.

According to the Kidney Disease Improving Global Outcomes (KDIGO) guidelines, AKI is defined by an increase in serum creatinine (within 2–7 days) and/or decrease in urinary output, while CKD is determined based on decreased eGFR < 60 mL/min/1.73 m^2^ and albuminuria persistent for more than 3 months [13]. The Fractional Excretion of Sodium (FeNa) and Fractional Excretion of Urea (FeUrea) are key tests for distinguishing prerenal from intrinsic renal AKI [45]. FeNa < 1% suggests prerenal AKI, while FeNa > 2% indicates intrinsic renal AKI. FeUrea < 35% suggests prerenal AKI, and FeUrea > 50% suggests intrinsic AKI, especially in patients on diuretics. Urine specific gravity and osmolality also aid in the diagnosis; high specific gravity (>1.020) and osmolality (>500 mOsm/kg) point to prerenal AKI, while low specific gravity (<1.010) and osmolality (<300 mOsm/kg) suggest intrinsic renal injury, such as acute tubular necrosis (ATN) [46].

In PwCF, data on urinary protein composition and concentration remain limited. However, recent studies have begun to shed light on renal involvement in CF. Notably, Rosner et al. found that total urinary protein, normalized to creatinine, was significantly elevated in PwCF compared to healthy controls [47]. Interestingly, this increase in proteinuria did not correlate with eGFR, and the urine albumin/creatinine ratio was similar between CF and control cohorts. These findings suggest that the elevated urinary protein observed in PwCF is not due to glomerular albumin leakage but may instead reflect subclinical tubular injury, potentially related to CFTR dysfunction in the renal epithelium.

### 2.4. Other Tests

Several other tests can provide valuable insight into kidney health. Urinalysis is a standard diagnostic tool used to evaluate kidney function and detect abnormalities in urine composition. The presence of red blood cells (RBCs) in urine (hematuria) can indicate glomerular disease or lower urinary tract abnormalities such as nephrolithiasis. Pyuria (the presence of white blood cells, WBCs) suggests urinary tract infection or interstitial nephritis. Cellular casts, including RBC casts, indicate glomerulonephritis, while WBC casts suggest interstitial nephritis or infection. Granular casts are frequently observed in ATN and indicate tubular injury, while hyaline casts, which can be found in concentrated urine or with dehydration, are less specific. Crystals in the urine can indicate metabolic disturbances and nephrolithiasis [48]. Kidney biopsy and histological evaluation of kidney tissue provide the most specific diagnosis and may predict the severity and prognosis of kidney disease. The kidney complications in PwCF have been recently reviewed [6]. Many of the standard clinical tests reviewed above are used for their diagnosis (Table 2) [6,49,50,51,52,53,54,55,56,57,58,59,60,61].

**Table 2:** Standard tests used in clinical practice for the diagnosis of common kidney complications in PwCF (modified from Hart et al.) [6].

Kidney Complication	Laboratory/Imaging Test for Detection
AKI	Serum creatinine, BUN, urinalysis, urine microscopy, FeNa, FeUrea, and urine output monitoring [49]
Pseudo-Bartter syndrome	Serum electrolytes, venous blood gas, and urine electrolytes [50]
AA amyloidosis	Serum amyloid A, kidney biopsy, and SAP scintigraphy [51,52]
IgA nephropathy	Urinalysis, urine microscopy, UPCR, and kidney biopsy [53]
Diabetic glomerulopathy	Urinalysis, UACR, eGFR, and kidney biopsy [54]
Tubulointerstitial nephritis	Urinalysis, kidney ultrasound, and kidney biopsy [55,56]
CKD	eGFR, UACR, UPCR, and urinalysis [57]
LMW proteinuria (non-glomerular)	Urine β2MG, and UPEP [58]
Hypercalciuria/ nephrolithiasis/ nephrocalcinosis	UCaCR, 24 h urinary analysis calcium excretion, urine microscopy, serum 25-hydroxyvitamin D, and kidney ultrasound [59,60,61]

AKI, acute kidney injury; BUN, blood urea nitrogen, FeNa, fractional excretion of sodium; FeUrea, fractional excretion of urea, AA amyloidosis, secondary amyloidosis; SAP, serum amyloid P component); IgA, immunoglobulin A; UPCR, urine protein to creatinine ratio; UACR, urine albumin to creatinine ratio; eGFR, estimated glomerular filtration rate; CKD, chronic kidney disease; LMW, low molecular weight; β2MG, beta-2 microglobulin; UPEP, urine protein electrophoresis; UCaCR, urine calcium to creatinine ratio.

## 3. Emerging Biomarkers of Early Kidney Injury in the General Population and PwCF

As described above, conventional tests report already-established kidney damage and are insensitive to subclinical or early injury. In contrast, novel biomarkers in the blood and urine may provide insights into the timing, severity, and site of kidney injury [62]. Molecular alterations revealed in genomic, proteomic, and metabolomic studies may recognize renal damage in the preclinical phase and provide insight into the pathophysiology of early kidney disease. Urinary biomarkers have already been recommended for detecting drug-induced tubular injury in early clinical trials [63].

Kidney injury molecule (KIM)-1 showed a strong negative correlation with eGFR in both AKI and CKD settings [64,65,66]. KIM-1 is a phosphatidylserine receptor expressed on the proximal tubules and targets apoptotic cells to lysosomes. Interleukin-18 (IL-18) and neutrophil gelatinase-associated lipocalin (NGAL) are also considered potential biomarkers for kidney injury [47,67,68]. The cell cycle arrest biomarkers tissue inhibitor of metalloproteinases-2 (TIMP-2) and insulin-like growth factor-binding protein 7 (IGFBP7) have emerged as promising indicators for detecting acute kidney injury (AKI), particularly in critically ill patients and those undergoing cardiac surgery. Recognizing its diagnostic potential, the NephroCheck™ test, which quantifies the product of [TIMP-2] × [IGFBP7], was approved by the U.S. Food and Drug Administration (FDA) in 2014 for clinical use in intensive care unit (ICU) settings to assess the risk of developing moderate to severe AKI [69]. Selenium-binding protein 1 (SBP1) plays a multifaceted role in cellular processes, exhibiting predominant expression in proximal tubular cells under normal conditions. Elevated urinary SBP1 levels have been identified as an early and sensitive biomarker of AKI, outperforming traditional markers like NGAL and TIMP-2 [70,71]. Kidney injury and inflammatory markers and tests across CKD stages are summarized in Table 3 [70,71,72,73,74,75,76,77,78,79,80,81,82,83,84,85].

**Table 3:** Kidney injury and inflammatory markers and tests across CKD stages. Levels of early kidney injury markers such as KIM-1, NGAL, and IL-18 increase first, while indicators such as serum creatinine, BUN, and Cystatin C increase in later stages.

Biomarker/Test	Type	Early Stage (Subclinical Injury)	Intermediate Stage (Functional Decline)	Late Stage (Established CKD)
Serum creatinine [70,71]	Functional decline	Normal	Starts increasing	High in late-stage CKD
BUN [72,73]	Functional decline	Normal	Increases moderately	High in severe CKD
Cystatin C [74,75]	Functional decline	Normal or slightly elevated	Moderately increased	High in severe CKD
eGFR [76]	Functional decline	Normal or even high	Progressive decline	Severely reduced in CKD
Proteinuria [77]	Glomerular dysfunction	Slight increase	Increases significantly	Severe elevation in CKD
KIM-1 [78]	Tubular injury marker	Early rise	Decreases with chronicity	Low but persists in CKD
β2MG [79,80]	Glomerular dysfunction	Normal or slightly elevated	Moderately increased	High in severe CKD
NGAL [81]	Tubular injury marker	Early and rapid increase	Fluctuates with injury	Persistent in progressive CKD
IL-18 [82,83]	Inflammatory marker	Early marker of tubular damage	Moderate increase	Can persist in CKD
TNFα [84,85]	Inflammatory marker	Low or normal	Elevated due to chronic inflammation	Persistently high in late CKD

CKD, chronic kidney disease; BUN, blood urea nitrogen; eGFR, estimated glomerular filtration rate; KIM-1, kidney injury molecule-1; β2MG, beta-2 microglobulin; NGAL, neutrophil gelatinase-associated lipocalin; IL-18, interleukin-18; TNFα, tumor necrosis factor α.

**Figure 1:**
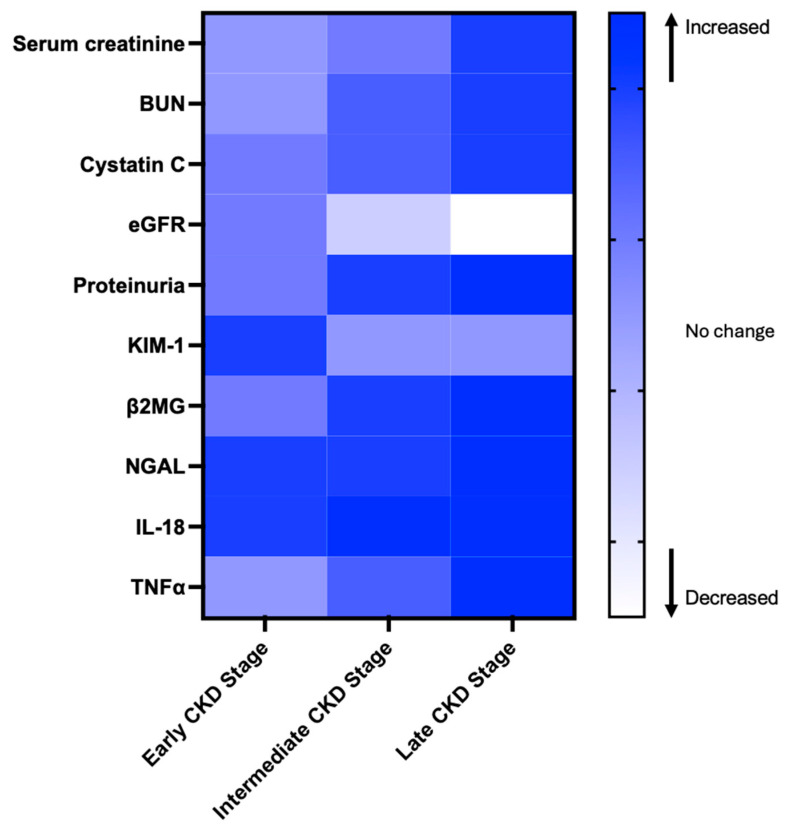
The heatmap illustrates changes in biomarker levels across different stages of chronic kidney disease (CKD). Color changes represent whether a biomarker is reported to increase or decrease, based on literature evidence. This figure is for visualization purposes only and does not reflect actual biomarker concentrations [70,71,72,73,74,75,76,77,78,79,80,81,82,83,84,85].

Even in the absence of albuminuria and with normal eGFR, PwCF exhibit evidence of both tubular cell dysfunction and renal injury, including decreased levels of urinary EGF (uEGF), and elevated levels of urinary tubular injury markers such as KIM-1, TFF3, β2MG, Cystatin C, and N-acetyl-β-D-glucosaminidase (NAG) [47]. These findings confirm that renal tubular injury occurs before significant glomerular dysfunction becomes evident, and decreased uEGF may suggest early signs of CKD in some PwCF [47,86,87]. Levels of KIM-1 and β2MG are also increased in PwCF during pulmonary exacerbations and correlate with lung function decline, pointing to the role of lung infection and inflammation in contributing to renal injury [47]. Increased urinary NAG levels suggest subclinical tubular kidney injury in PwCF undergoing aminoglycoside therapy, including nebulized tobramycin in children [88,89]. Elevated urinary soluble Fas (sFas) concentrations during aminoglycoside treatment are associated with the development of CKD in PwCF [90]. While these observations were limited to a few tubular injury markers, which have yet to be systematically studied in PwCF, they support the concept of a novel tool for the rapid and early detection of kidney injury before elevation of serum creatinine or urinary protein. Changes in markers and tests levels across CKD stages are summarized in Figure 1.

Extracellular vesicles (EVs) are a source of emerging site- and disease-specific kidney injury markers [91]. EVs are membrane-bound nanosized particles released by cells that play a significant role in intercellular communication by transferring signals capable of altering target cell function and influencing the pathophysiology of various diseases, including CF [92]. Urinary extracellular vesicles (uEVs), which are EVs present in urine, have been shown to mediate the crosstalk between different cell populations during nephrogenesis, amplification of kidney injury, inflammation, fibrosis, and regeneration [91]. Intercellular communication in the kidney occurs not only between mesangial, endothelial, and podocyte cells within the glomeruli, but also between glomerular and tubular compartments and among different tubular cell types [93].

Both the number of uEVs released and their contents (proteins, lipids, DNA, and RNA species) change under cellular stress and have been investigated as biomarkers for kidney disease [94]. It has been proposed that the characteristic signatures of the EV cargo may be leveraged as markers for location-specific kidney disease [95,96,97]. Because AKI frequently involves acute tubular damage that can progress to chronic injury characterized by interstitial fibrosis, the crosstalk between the tubular and interstitial compartments has become a key focus for uncovering new mechanisms underlying maladaptive repair processes and the progression from AKI to CKD [93]. A panel of proteins and micro(mi)RNAs have been associated explicitly with AKI versus CKD, glomerular versus tubular renal injury, specific kidney disease processes, and maladaptation after AKI [91]. Thus, elevated levels of specific EV markers may provide mechanistic information, and an early index of AKI may allow for monitoring of CKD progression in PwCF. However, further research is needed to validate these markers in PwCF and to explore their application in early diagnosis and monitoring of kidney disease progression.

Altered levels of specific proteins in uEVs have been linked to kidney injury. NGAL in uEVs, but not in free urine, leads to increases in delayed graft function post-transplant, indicating EVs may protect and enrich certain biomarkers [98]. Panich et al. identified exosomal activating transcription factor-3 (ATF3) as an early marker of sepsis-induced AKI [99]. Increased aquaporin-2 (AQP2) in uEVs signals collecting duct injury after transplantation, while low preoperative podocalyxin levels suggest early podocyte damage and risk of postoperative AKI [100,101]. Decreased CD133 (Prominin-1), a renal progenitor cell marker, in uEVs is seen in both AKI and CKD, reflecting reduced regenerative capacity [102]. MicroRNAs such as miR-125a-5p and miR-10a-5p are also reduced in uEVs of patients who later develop severe AKI, highlighting their potential in risk stratification [101]. Classical exosomal markers CD9 and CD63 are often elevated in uEVs of kidney transplant recipients with AKI, indicating increased vesicular trafficking during injury [100]. Wilms tumor-1 (WT-1), essential for podocyte integrity, is detected in uEVs as a marker of glomerular damage in various kidney diseases [103]. In chronic kidney injury, p16^INK4a^, a cellular senescence marker, is found in uEVs and linked to hypertensive nephropathy, providing insights into renal aging and fibrosis [104]. Takizawa et al. used a Tim4-based ELISA to measure uEV markers, including distal tubule/collecting duct-specific Mucin 1 (MUC1) and proximal tubule-specific maltase-glucoamylase (MGAM), showing the MGAM/MUC1 ratio rises as kidney function declines in CKD patients [105]. Together, these uEV-associated markers provide valuable insights into molecular events in AKI and CKD and show promise for precision nephrology. However, they have not yet been validated in PwCF.

### Clinical Utility of Biomarkers in CFKD

Several biomarkers discussed in this review demonstrate varying clinical applicability in kidney injury. Traditional markers such as serum creatinine, BUN, and eGFR are routinely used in clinical practice to assess kidney function and are applicable to CFKD. However, have limited sensitivity for detecting early kidney injury. Cystatin C, a low molecular weight protein less influenced by muscle mass, has also been increasingly utilized in clinical settings for more accurate eGFR estimation Proteinuria and β2-microglobulin (β2MG) are established but not disease-specific indicators of kidney dysfunction and may aid in detecting renal involvement in CF. Emerging tubular injury markers like KIM-1 and NGAL have demonstrated potential for identifying early subclinical injury and could help monitoring CFKD progression, though they are not yet standard in clinical care. Similarly, inflammatory markers such as IL-18 and TNFα are being studied for their potential to detect ongoing kidney inflammation in CF. Together, these biomarkers provide a foundation for understanding and potentially stratifying CFKD progression. However, broader clinical adoption of newer candidate markers will require further validation in CF-specific patient cohorts.

## 4. Potential Genetic Modifiers of CFKD in PwCF

CKD and CF share an extensive literature regarding genetic modifiers of disease progression, with potential overlapping mechanisms. Although the genetic modifiers influencing disease severity have been extensively studied and separately validated in CF and CKD, it remains unknown how the modifiers associated with CF lung disease or CF-related diabetes affect the risk of CF-related kidney disease [106,107,108,109]. It is also unclear whether the risks of CKD progression identified in the general population have a similar effect in PwCF. Below, we review several gene polymorphisms associated with a potential for influencing the risk and severity of CKD in PwCF (Table 4).

Transforming growth factor β1 (TGFβ1) is an established genetic modifier in CF. Several TGFβ1 polymorphisms are associated with CF progression and *P. aeruginosa* infection [110]. Elevated TGFβ1 levels downregulate CFTR mRNA levels through miR-145-5p [111]. In addition to CFTR, TGFβ1 also regulates Calcium-activated Chloride Conductance (CaCC), thereby maintaining normal hydration of epithelial surfaces, including the airways and colon [112]. Aberrant TGFβ1 signaling drives HNF1B-Related Autosomal Dominant Tubulointerstitial Kidney Disease characterized by tubular cysts, renal fibrosis, and progressive decline in kidney function [113]. Genetic variations in TGFβ1 are also associated with susceptibility to IgA nephropathy [114], autosomal dominant polycystic kidney disease (ADPKD) [115], and CKD [116]. Furthermore, IgA nephropathy is the most common glomerular disease as one of the kidney complications in PwCF [6].

**Table 4:** Common genetic determinants in CF and CKD.

Modifier	Role in PwCF	Role in CKD
TGFβ1	The most established genetic modifier in CF. Several TGFβ1 polymorphisms are associated with CF progression and *P. aeruginosa* infection [110].	Drives HNF1β-induced ADTKD [113]. Susceptibility to IgAN [114], polycystic kidney diseases [115], and CKD [116].
ACE	The D/I polymorphism is associated with disease severity [117]. Expression and localization are controlled by CFTR [118,119].	DD genotype is a risk factor for CKD [120]. ID/DD genotypes are associated with chronic lesions, such as capsular adhesions or glomerulosclerosis and proteinuria in severe IgAN [121].
MBL2	Decreased survival and increased susceptibility to infections to *P. aeruginosa* and worse lung functions [122,123]. The pathogenic variants are Gly54Asp (the B allele, rs1800450), Gly57Glu (the C allele, rs1800451), and Arg52Cys (the D allele, rs5030737), which together referred to as 0 allele [124].	Glomerular deposition of MBL has been consistently observed in kidney biopsy specimens in people with IgAN [125,126]. High serum levels are also associated with the development and progression of diabetic nephropathy [127].
AAT	The most abundant proteinase inhibitor in the lung with anti-inflammatory effects. Genetic modifier protecting against disease progression [128,129,130,131]. M (normal), S (264Glu → Val) and Z (342Glu → Lys) [132]. Contradictory results for the effect of S and Z alleles [130,132,133,134].	Rapid rise in serum levels predicts AKI in experimental and clinical settings [135,136]. S and Z alleles were associated with high levels of the antigen of ANCA in Granulomatosis with polyangiitis [137]. In CKD, AAT has a protective effect [138].
β2AR	Stimulation results in improved lung function [139]. The Gly^16^Glu^27^ genetic variant upregulates CFTR activity [140,141].	Expressed in proximal tubules, glomeruli, and podocytes [142]. Anti-inflammatory [143].
TNFα	(-308 GA, rs1800629) polymorphism is associated with CF [124]. +691g ins/del polymorphic locus is associated with the severity of lung disease and. aeruginosa infection [144]. TNFα -308GA promoter polymorphism (rs1800629) that were associated with high TNFα transcription, CF and AKI severity [145,146,147].	High levels disrupt the localization of PC2 to the plasma membrane and primary cilia in ADPKD [148,149].
IL-10	Anti-inflammatory cytokines present at low levels in PwCF. The haplotype GCC/ACC is significantly associated with *P. aeruginosa* infection and CF severity [150]. A significant association was found between the −1082GG genotype and colonization with *A. fumigatus* and allergic bronchopulmonary aspergillosis [151].	Important role in normal physiology, AKI and CKD progression [152]. Polymorphisms are associated with AKI [147]. L-10 -1082 A/G polymorphism was associated with an increased risk of AKI [153] and primary glomerulonephritis [154].
NOS	Low levels of exhaled NO [155,156]. NOS1 and NOS2 polymorphisms are associated with disease severity and inflammation [157,158]. G847T polymorphism in the *NOS3* gene, is associated with high NO production had a slower decline in lung function [159,160].	Levels are reduced in CKD. NOS inhibition causes systemic and glomerular hypertension, glomerular ischemia, glomerulosclerosis, tubulointerstitial injury, and proteinuria [161]. Presence of the two *NOS3* gene polymorphisms, Glu298Asp polymorphisms 4 b/a and -786T > C is a risk of ESKD in patients with CKD and ADPKD [162,163].
GST	M1 (GSTM1) allele associated with worse lung disease [164]. GSTM3*B allele contributes to clinical severity in CF [165].	GSTM1, GSTT1, and GSTP1 polymorphisms are risk of ESKD [166]. GSTM1 deletion is associated with more rapid progression of pediatric CKD [167].

TGFβ1, transforming growth factor β1; CF, cystic fibrosis; HNF1β, hepatocyte nuclear factor 1-beta; ADTKD, autosomal dominant tubulointerstitial kidney disease; IgAN, immunoglobulin A nephropathy; ACE, angiotensin-converting enzyme; CFTR, CF transmembrane conductance regulator; MBL2, mannose-binding lectin; AAT, α1-antitrypsin; AKI, acute kidney injury; ANCA, antineutrophil cytoplasm antibodies; CKD, chronic kidney disease; β2AR, beta-2-adrenergic receptor; TNFα, tumor necrosis factor α; PC2, polycystin 2; ADPKD, autosomal dominant polycystic kidney disease; IL-10, interleukin-10; PwCF, people with cystic fibrosis; NOS, nitric oxide synthase; NO, nitric oxide; ESKD, end-stage kidney disease; GST, glutathione S-transferase.

TNFα-308 GA polymorphism is associated with CF in diverse populations [124]. The TNFα +691g ins/del polymorphic locus is associated with the severity of CF lung disease and the age of onset of *P. aeruginosa* infection [144]. Different studies have found TNFα-308 GA promoter polymorphism (rs1800629) that was associated with high TNFα transcription, CF, and AKI severity [145,146,147]. High levels of TNFα induce scaffold protein FIP2, which disrupts the localization of polycystin 2 (PC2) to the plasma membrane and primary cilia in ADPKD [148,149].

Angiotensin-converting enzyme (ACE) is an enzyme in the renin–angiotensin–aldosterone system that is essential for regulating blood pressure and fluid balance. *ACE* gene D/I polymorphism is a modulator of the severity of CF [117]. ACE2 expression and localization are regulated by the *CFTR* gene, suggesting a possible role in the progression of CF disease [118,119]. ACE also regulates kidney function, and ACE inhibitors are commonly used to treat kidney disease [168]. Among hypertensive patients, the ACE-DD genotype has been shown to be a risk factor for the causation and development of chronic kidney failure [120]. In severe forms of IgA nephropathy (IgAN), ID/DD genotypes are associated with chronic lesions, such as capsular adhesions or glomerulosclerosis and proteinuria [121].

Mannose-binding lectin (MBL2), a member of C-type lectin family activates the complement system during inflammation and can cause both pathogen clearance and tissue injury [166]. In CF, low MBL2 levels are associated with decreased survival and increased susceptibility to *P. aeruginosa* infection and worse lung function [122,123]. A non-linear association between MBL levels and renal outcome has been found in IgAN, with both MBL deficiency and excess independently linked to poor renal outcomes, suggesting that MBL contributes to IgAN progression through multiple mechanisms [125,126]. High serum MBL levels are also associated with the development and progression of diabetic nephropathy [127].

α1-antitrypsin (AAT) is the most abundant proteinase inhibitor within the lung and prevents tissue damage caused by inflammation. AAT is considered a genetic modifier in CF, and exogenous AAT has been proposed as a potential therapy for CF [128,129]. However, two separate clinical studies suggest a protective effect of low to moderate levels of AAT on the progression of CF [130,131]. There are three alleles for AAT: M (normal), S (264Glu → Val), and Z (342Glu → Lys). The prevalence of S and Z alleles is approximately 12% in the CF population. Homozygous S and Z alleles result in 60% and 10% of plasma AAT levels in the homozygous state when compared with the homozygous M allele [132]. In CKD, AAT has a protective effect [138]. Two studies showed contradictory results regarding the effect of S and Z alleles on the age of onset of chronic *P. aeruginosa* acquisition in patients with CF [133,134]. Another polymorphism in the 3′UTR of AAT (G1237A) is associated with a small rise in AAT levels during the acute inflammatory conditions in CF [132], although a later study with a large sample size found no association between G1237A and lung functions in CF patients [130]. A rapid rise in AAT levels can predict AKI in experimental and clinical settings [135,136]. S and Z alleles have been associated with high levels of the antigen of antineutrophil cytoplasm antibodies (ANCA) in Granulomatosis with polyangiitis [137]. In conclusion, despite the considerable amount of data, the impact of AAT on CF phenotype is still unclear, and its role in CF and CKD warrants further investigations.

It has been shown that stimulation of β-adrenoceptor (β-AR) results in increased alveolar fluid clearance and ciliary beat frequency in PwCF [139]. The Gly16Glu27 β2AR genetic variant upregulates CFTR activity in adult CF patients [140]. A similar effect is observed with β2AR agonist salbutamol and ritodrine, suggesting that the effect increases β2AR activity [141]. In the kidney, β2AR is expressed in proximal tubules, glomeruli, and podocytes [142]. Similarly to its role in CF, β2AR agonist reduces the proinflammatory response to renal inflammation [143].

Interleukin-10 (IL-10) is an anti-inflammatory cytokine present in low levels in CF patients. In a study of 220 CF patients, IL-10 haplotype GCC/ACC was significantly associated with *P. aeruginosa* infection and CF severity [150]. In another study of 378 patients with CF, a significant association was found between the −1082GG genotype and colonization with *A. fumigatus* and allergic bronchopulmonary aspergillosis [151]. IL-10 plays an important role in renal physiology, AKI, and progression of chronic renal failure [152]. Polymorphism in IL-10 is associated with AKI (AKI) [147]. IL-10-1082 A/G polymorphism was associated with increased risks of AKI [153] and primary glomerulonephritis [154].

Nitric oxide synthase (NOS) is an immune modulator and vasodilator. PwCF typically show below normal levels of exhaled NO [155,156]. NOS1 and NOS2 polymorphisms are associated with disease severity and inflammation. More specifically, the number of AAT repeats in the *NOS1* gene is negatively correlated with nasal and expired NO in PwCF and positively correlated with *P. aeruginosa* and *A. fumigatus* infection [157,158]. Separate studies have found that the number of GT repeats in NOS1 promoter and G847T polymorphism in the *NOS3* gene are positively associated with increased NO production and slower decline in lung function [159,160]. NO levels are reduced in CKD [169]. Experimental NOS inhibition is associated with systemic and glomerular hypertension, glomerular ischemia, glomerulosclerosis, tubulointerstitial injury, and proteinuria [161]. Meta-analysis of 13 studies suggests the presence of the two *NOS3* gene polymorphisms, Glu298Asp polymorphisms 4 b/a and -786T > C that have been associated with an increased risk of ESKD in patients with CKD and ADPKD [162,163].

Glutathione S-transferase (GST) is an antioxidant enzyme that conjugates hydroperoxides with glutathione, thereby mitigating tissue damage. CF patients homozygous for glutathione S-transferase M1 (GSTM1) allele have worse chest radiographic scores and worse Shwachman–Kulczycki (SK) scores of CF disease severity [164]. Another polymorphism of the GSTM3*B allele contributes to clinical severity in CF [165]. A meta-analysis found an association between GSTM1, GSTT1, and GSTP1 genetic polymorphisms and the risk of ESKD [166]. Another study of 674 children identified the association of GSTM1 deletion with more rapid progression of pediatric CKD [167]. In conclusion, GWAS studies are needed to examine the role of specific CF genetic modifiers in CFKD risk.

ß-catenin has emerged as a key contributor to kidney fibrosis in the context of CFTR dysfunction. Zhang et al. showed a clear upregulation of the ß-catenin pathway in renal epithelial cells with CFTR knockdown and in the kidneys of F508del mice subjected to unilateral ureteral obstruction (UUO), a well-established model of kidney fibrosis [17]. Furthermore, knockdown of CFTR reduced the expression of tight junction proteins Occludin and ZO-1 in kidney tubular cells, reasoning that it could be a cause of increased leakage of low molecular weight proteins as seen in the urine of PwCF. This, along with enhanced epithelial-to-mesenchymal and fibrosis seen in CFTR mutant mice, may underlie the frequent kidney disease observed in CF.

## 5. Future Directions of Research on Kidney Function in PwCF

Elucidating the mechanisms of kidney injury is essential for its early detection and prevention. A critical question in CFKD is to what extent the increased prevalence of AKI and CKD results from extrarenal causes, including CF lung disease and nephrotoxic drug exposure, as opposed to intrinsic susceptibility to tubulointerstitial or glomerular injury resulting from renal CFTR dysfunction, decreased nephron endowment [170], pH sensitivity, or pro-fibrotic signaling. Although many of these pathways have been observed in human biospecimens, separating a primary defect from the response to injury is difficult. The recent development of excellent CF animal models [171,172,173] may allow for in vivo distinction of the effect of CFTR dysfunction on kidney health, as well as on the impact of CFKD on the aging population of PwCF in the era of HEMT. These models are increasingly responsive to clinically relevant CFTR modulators [174,175], helping to distinguish the response to nephrotoxic injury on or off therapy. For example, the humanized G551D CF rat model may prove a valuable tool for studying CF kidney disease, as it spontaneously develops lung disease [176], responds to the CFTR modulator Ivacaftor [174], and offers an appealing husbandry profile for reproduction and cost. Similarly, CFTR-knockout ferrets, which mimic human lung and pancreatic disease, offer a promising model to study CFTR-related kidney damage [177]. Additionally, machine learning presents a promising approach with the potential to identify PwCF at risk for kidney disease. Machine learning algorithms are capable of predicting the onset of CKD in asymptomatic individuals, improving risk stratification for progression or complications, and identifying distinct kidney phenotypes or subtypes by linking clinical features to underlying pathological mechanisms [178]. In the context of CF-related kidney disease, machine learning could be applied to integrate multi-dimensional datasets—including urinary and blood biomarkers, genotype (e.g., CFTR variants), comorbidities (such as CFRD), and medication exposure—to identify early signs of renal involvement and personalize treatment strategies. These tools could also help prioritize candidate biomarkers for clinical use and uncover patterns not readily apparent through conventional analysis. Incorporating machine learning into future CFKD studies may enhance our ability to deliver precision nephrology in this vulnerable population.

## Abbreviations

The following abbreviations are used in this manuscript:

CF	Cystic Fibrosis
CFTR	CF Transmembrane Conductance Regulator
HEMT	Highly effective modulator therapy
PwCF	People with CF
CKD	Chronic kidney disease
CFKD	CF-related kidney disease
ESKD	End-stage kidney disease
AKI	Acute kidney injury
GFR	Acute kidney injury
eGFR	Estimate GFR
IDMS	Isotope dilution mass spectrometry
HPLC	High-performance liquid chromatography
CKiD	Chronic Kidney Disease in Children
CKD-EPI	Chronic kidney disease epidemiology collaboration
MDRD	Modification of diet in renal disease
UACR	Urine albumin to creatinine ratio
LMW	Low molecular weight
β2MG	Beta-2 microglobulin
RBP	Retinol-binding protein
ELISA	Enzyme-linked immunosorbent assay
SDS-PAGE	Sodium dodecyl sulfate-polyacrylamide gel electrophoresis
LC-MS	Liquid chromatography-tandem mass spectrometry
UPEP	Urine protein electrophoresis
KDIGO	Kidney Disease Improving Global Outcomes
FeNa	Fractional excretion of sodium
FeUrea	Fractional excretion of urea
ATN	Acute tubular necrosis
RBC	Red blood cell
WBC	White blood cell
BUN	Blood urea nitrogen
SAP	Serum amyloid P component
IgA	Immunoglobulin A
UPCR	Urine protein to creatinine ratio
UCaCR	Urine calcium to creatinine ratio
KIM-1	Kidney injury molecule-1
IL-18	Interleukin-18
TIMP-2	Tissue inhibitor of metalloproteinases-2
IGFBP7	Insulin-like growth factor-binding protein 7
FDA	Food and Drug Administration
SBP1	Selenium-binding protein 1
NGAL	Neutrophil gelatinase-associated lipocalin
TNFα	Tumor necrosis factor α
NAG	N-acetyl-β-D-glucosaminidase
sFas	Soluble Fas
EVs	Extracellular vesicles
uEVs	Urinary extracellular vesicles
ATF3	Activating transcription factor-3
AQP2	Aquaporin-2
CD133	Prominin-1
WT-1	Wilms tumor-1
MUC1	Mucin 1
MGAM	Maltase-glucoamylase
TGFβ1	Transforming growth factor β1
HNF1β	Hepatocyte nuclear factor 1-beta
ADTKD	Autosomal dominant tubulointerstitial kidney disease
IgAN	Immunoglobulin A nephropathy
ACE	Angiotensin-converting enzyme
MBL2	Mannose-binding lectin
AAT	α1-antitrypsin
ANCA	Antineutrophil cytoplasm antibodies
β2AR	Beta-2-adrenergic receptor
PC2	Polycystin 2
ADPKD	Autosomal dominant polycystic kidney disease
IL-10	Interleukin-1
NOS	Nitric oxide synthase
NO	Nitric oxide
GST	Glutathione S-transferase
CaCC	Calcium-activated chloride conductance
GSTM1	Glutathione S-transferase M1
SK	Shwachman–Kulczycki
